# Ontogeny of Foraging Competence in Capuchin Monkeys (*Cebus capucinus*) for Easy versus Difficult to Acquire Fruits: A Test of the Needing to Learn Hypothesis

**DOI:** 10.1371/journal.pone.0138001

**Published:** 2015-09-15

**Authors:** Elizabeth Christine Eadie

**Affiliations:** School of Anthropology, University of Arizona, Tucson, Arizona, United States of America; Texas A&M University, UNITED STATES

## Abstract

Which factors select for long juvenile periods in some species is not well understood. One potential reason to delay the onset of reproduction is slow food acquisition rates, either due to competition (part of the ecological risk avoidance hypothesis), or due to a decreased foraging efficiency (a version of the needing to learn hypothesis). Capuchins provide a useful genus to test the needing to learn hypothesis because they are known for having long juvenile periods and a difficult-to-acquire diet. Generalized, linear, mixed models with data from 609 fruit forage focal follows on 49, habituated, wild *Cebus capucinus* were used to test two predictions from the needing-to-learn hypothesis as it applies to fruit foraging skills: 1) capuchin monkeys do not achieve adult foraging return rates for difficult-to-acquire fruits before late in the juvenile period; and 2) variance in return rates for these fruits is at least partially associated with differences in foraging skill. In support of the first prediction, adults, compared with all younger age classes, had significantly higher foraging return rates when foraging for fruits that were ranked as difficult-to-acquire (return rates relative to adults: 0.30–0.41, p-value range 0.008–0.016), indicating that the individuals in the group who have the most foraging experience also achieve the highest return rates. In contrast, and in support of the second prediction, there were no significant differences between age classes for fruits that were ranked as easy to acquire (return rates relative to adults: 0.97–1.42, p-value range 0.086–0.896), indicating that strength and/or skill are likely to affect return rates. In addition, fruits that were difficult to acquire were foraged at nearly identical rates by adult males and significantly smaller (and presumably weaker) adult females (males relative to females: 1.01, p = 0.978), while subadult females had much lower foraging efficiency than the similarly-sized but more experienced adult females (subadults relative to adults: 0.34, p = 0.052), indicating that skill, specifically, is likely to have an effect on return rates. These results are consistent with the needing to learn hypothesis and indicate that long juvenile periods in capuchins *may* be the result of selection for more time to learn foraging skills for difficult-to-acquire fruits.

## Introduction

Despite a considerable amount of research on the topic, the main factors that select and have selected for long juvenile periods in primates compared with other mammals, and humans compared with other primates, remain only partially understood and under considerable debate [1–4]. The needing to learn hypothesis provides one potential explanation for this trend in certain primate taxa [5–7]. According to this hypothesis, complex and unpredictable niches select for both an increase in brain size and an increase in the length of the juvenile period, because these traits enable organisms the additional time, and a greater ability, to learn how to solve problems in their environments [5]. One version of the needing to learn hypothesis posits that reliance on a difficult-to-acquire diet selects for longer juvenile periods [8–10]. From here on I will refer to this version of the needing to learn hypothesis as the difficult diet hypothesis.

Critics of the difficult diet hypothesis point out that studies have failed to find a correlation between foraging complexity, age at first reproduction, and brain size across primates [5], that juveniles achieve adult-levels of foraging efficiency well before the onset of reproduction [11, 12], and that differences in efficiency between adults and juveniles are better explained by strength rather than skill [13]. Others have pointed out that the correlation between the length of the juvenile periods and foraging complexity is not necessarily predicted by the needing to learn hypothesis when applied to large, unspecified datasets, since it is likely to apply to some species and not others [6, 14]. A recent, comparative study found that the needing to learn hypothesis for long juvenile periods remains viable for specific species [6]. In addition, correlations between the time needed to learn skills and the length of the juvenile period are only expected for the most difficult/complex aspects of an organisms niche [7, 14]. The lack of a correlation between diet categories and the onset of reproduction should be expected if species that do not inhabit complex niches have brain sizes and juvenile periods set by energetic constraints (see [6]), ecological risk avoidance (see [11]), and/or maturational constraints (see [14]) rather than time to learn. And in fact, a number of researchers have found that needing to learn and/or ecological risk avoidance do not seem to explain prolonged juvenescence in a number of species [15–19]. This does not rule out the possibility that either or both of these hypotheses are viable for other species, but it does highlight the complexity of factors that determine growth rates and timing of reproduction across species.

The embodied capital theory of evolution provides a general framework for understanding which niches should select for long juvenile periods and big brains, dependent on how that organism makes a living [20, 21]. By considering how time and energetic investments into various types of embodied capital (e.g. skeletal structure, muscle, brain growth, skill acquisition, maintenance of social bonds, etc.), may impact an organism’s evolutionary fitness, this theory predicts that organisms living in environments that are more complex and less predictable should invest more heavily in certain types of embodied capital. For example, in niches where organisms benefit from a greater degree of skill and comprehension, investments into embodied capital in the forms of a) brain tissue, which can be used to respond to environmental challenges [20], and b) the development of specific skills [21, 22], will provide higher payoffs later in life. This strategy then has implications for life history traits, which can include selection for longer juvenile periods and lifespans [5, 10].

Capuchin monkeys (*Cebus spp*. and *Sapujus spp*.) have extraordinarily long juvenile periods [23, 24], and large brains [25] compared with other primates (relative to body size). Capuchins are also known for their complex dietary niche [26], which in certain species regularly involves tool use [27]. Capuchin diets are remarkable in that (a) they contain a large amount of animal protein for a monkey of capuchin size [28], (b) they can include relatively large vertebrates such as squirrels and nestling coatis [29], and (c) when foraging on plant matter, capuchins rely heavily on the nutrient dense, often protected storage and reproductive organs of plants [9, 26]. Many of the plant and animal foods that capuchin monkeys are known to exploit have elaborate defense mechanisms such as items with spines or thorns, biting or stinging insects, or hard shells [26, 30, 31]. These characteristics indicate that the difficult diet hypothesis provides a plausible explanation for the evolution of a long juvenile period in capuchin monkeys.

This study provides an empirical test of the difficult diet version of the needing to learn hypothesis by examining how the skill level necessary for acquiring and processing a fruit affects age at which adult foraging rates are achieved, in capuchin monkeys. The difficult diet hypothesis relates to the embodied capital model of evolution in that support for this hypothesis would indicate that capuchins inhabit an ecological niche that selects for increased embodied capital in the form of foraging skills. A critical prediction of the difficult diet hypothesis is that individuals delay reproduction to learn foraging skills. Only the food items that require the highest levels of skill to obtain are predicted to require at least the length of juvenile period to achieve maximum return rates. Therefore, the specific predictions of the difficult diet hypothesis tested in this study were: 1) pre-reproductive individuals have significantly lower foraging return rates for difficult-to-acquire food items compared with adults, and 2) higher foraging return rates are associated with a higher level of skill.

## Methods

Study site and permits: This study was conducted at the Pacuare Nature Reserve in the Limón Province of Costa Rica. The reserve is located 25 km north of the capital Puerto Limón, between the Tortuguero Canals and the Caribbean Sea (10°10’N, 83°14W). It contains 800 ha of mixed primary and secondary, tropical, wet, lowland forest. The Pacuare Nature Reserve is part of an international organization named the Endangered Wildlife Trust. Permission to collect data at this site was granted by Carlos Fernandez Alfaro, the general director of the Costa Rica and Panama Projects for the Endangered Wildlife Trust. In addition, a permit to collect data in Costa Rica was applied for and received every six months for the duration for the duration of the project through the MINAET office in San Jose, in accordance with Costa Rican government regulations.

Study groups: Data was collected on individuals within three groups of capuchins that have territories in the southern portion of the reserve. Their habitat consists of secondary, primary, and swamp lowland forest habitats. A total of seven and a half months were spent on identification and habituation of the three study groups, “A”,”B” and “C”, between 2005 and 2009, before the onset of this study. Body size, sex, and unique physical features such as the shape of the cap line and scars were used to identify each individual. Prior to the start of this study each study individual could be reliably identified by the author. Data were collected on 49 individuals within the three study groups.

Subjects included 6 infants (age 0–<1 year), 5 younger juveniles (ages 1–<3 years), 5 older juveniles (ages 3–<5 years), 6 subadults (ages 5–<7 years) and 27 adults (7 years and older). Age classes were defined in a similar way to other studies that examine foraging in juvenile capuchins [32, 33] and were designed to reflect differences in experience, size, and reproductive activity. In the wild, the average age at first reproduction for *Cebus capucinus* is seven years at Santa Rosa National Park [34], and 6.22 years at Lomas Barbudal [35]. In this study females were classified as adults if they were estimated to be greater than 7 years old or if they had given birth. To maintain a similar level of experience for the adult age class, males were also classified as adults if they were estimated to be greater than 7 years old, although males do not achieve adult size for several more years [36].

Several methods were used to determine the age class of subjects. First, when subjects were observed during an early phase of infancy (while still riding on the mother’s neck), the earliest sighting of that individual was designated as his/her birth month. For juvenile and subadult subjects who were not observed in early infancy, ages were estimated by comparing the earliest dated pictures of them with the chronological pictures of individuals of known ages. Individuals younger than age 5.5 years at the start of the study period (October 2009) would have been observed within their first year of life during the habituation period, and are therefore the most likely to be assigned ages that would be accurate to within 6 months. With increasing age, the uncertainty of age increases.

Data collection: Data for this study were collected between October 2009 and August 2010. Focal observations (“focal follows”) involved recording the duration of all behaviors exhibited by a focal individual, who was engaging in foraging activities, for a continuous period of time [37]. Individuals were selected for focal follows based on visibility and were only sampled once per food item per day. As many different individuals as possible were sampled each day. Focal follows lasted for the duration of a foraging event on one food type; if the focal individual stopped foraging or changed to a different food item the focal follow was ended. A foraging event began when a focal individual started to search for a food item. Subjects were chosen based on visibility and the food item being foraged. Only focal follows in which it was possible to obtain a reliable count of the number of bites that the focal individual took during the follow were included in the dataset. When a monkey moved out of sight during ingestion the focal follow was discarded. During focal follows, I narrated behaviors while my field assistant recorded them in real time on an HP iPaq using Noldus Observer 8.0 and Pocket Observer 3.1 behavioral data collection software (Noldus Information Technology, Wageningen, Netherlands). Inter- and intra- observer reliabilities were measured by comparing focal follow entries created from digital voice recordings of sample focal follows. Data were kept after a ≤1 second discrepancy in foraging behavior timing was achieved per test follow (duration: three-five minutes). As a preliminary control for access to a particular food resource, focal follows were only included in the dataset if aggression and displacements did not occur (i.e. subject access to food items was not obviously inhibited by social interactions).

In addition to focal follows, Scan samples [37] were conducted every 30 minutes while in the presence of a study group. Data from scan samples were used to calculate time allocation. Scan samples lasted for 5 minutes during which time the first behavior observed for each individual positively identified over the interval was recorded.

Return rates: Return rates for this study were computed as the number of bites swallowed (bites that were spit out were not included in the count), divided by the time the individual took to search, harvest, and process his/her food items (”total forage time”). Search was defined as the visual, olfactory, and/or manual investigation of potential food sites and items. Harvest was defined as the removal of a food item from a substrate. Process was defined as the manipulation of a food item that an individual already had in his/her possession, in order to improve ingestion or digestibility.

Food difficulty levels: Food items were assigned both a skill and a strength difficulty level for each of the three forage components: search, harvest, and process. Strength levels were categorized by whether no (strength level = 1), moderate (strength level = 2”, or intense (strength level = 3) force was necessary to complete the behavior. Intense force was defined as force that required leveraging, using body positions or use of an object, by individuals who were near or at adult body weight (older juveniles, subadults, and adults). Examples of foraging behaviors that require intense force include tearing apart canes in search of embedded larvae or pounding open a hard-shelled fruit on a branch. A moderately forceful manipulation was defined as one that required solely manual pressure by older juveniles, subadults, and/or adults. Examples include removing a fruit from a thick stem or removal of a thin, hard shell. For skill, the difficulty classification was based on whether zero (skill level = 1), one (skill level = 2), or greater than one (skill level = 3) skillful manipulation were necessary to complete the behavior. A skillful manipulation was defined as an action that requires one of the following: a dexterous manipulation, or a sequence-specific, condition-specific, or location-specific action. Examples include avoiding defense systems such as thorns or spikes, and locating larvae within canes. Although this method of difficulty level assignment was ordinal and thus less specific and more subjective than strictly quantitative measurements, it provides an easy and practical system for separation of foraging behaviors into strength and skill difficulty levels. The value for total strength was computed by taking the sum of search strength, harvest strength, and process strength, while the value for total skill was computed by taking the sum of search skill, harvest skill and process skill. Total difficulty is equal to the sum of total strength and total skill (see S1 Table for a description of search, harvest, and process requirements for the top ten most commonly eaten food items, and their associated strength and skill, and total difficulty level assignments; see S2 Table for the difficulty classification of all study foods). All food items in this dataset were successfully acquired by each age-sex category and, thus, strength levels are designed to reflect the extra time it might take a weaker individual to acquire a food item, rather than whether an individual is capable of obtaining a particular food item. Finally, in order to compare foods that were relatively difficult to acquire with foods that were relatively easy to acquire, foods were split into three categories (roughly thirds) based on their total difficulty score. Foods that had scores in the highest range were categorized as *difficult to acquire*, while foods that had scores in the lowest range were categorized as *easy to acquire*.

Data analyses: Statistical analyses were carried out in R v. 2.11.1 [38] using the lme4 package [39]. Generalized linear mixed models (GLMMs) were used to examine the effects of sex, age class, and difficulty levels on foraging return rates. Sex, a potential covariate, was added to each model as a fixed effect but removed if insignificant. Subject identity and food item were included in the model as random effects in order to control for repeated sampling and variability between food items within a difficulty level. Mixed effect models were chosen because they provide the best fit for non-normal, longitudinal data that include repeated measures of subjects [40].

Two tests examined the effect of strength vs. skill on foraging rates for difficult-to-acquire fruits. The first test examines the effect of sex on adult foraging efficiency for difficult-to-acquire fruits. Given that adult white-faced capuchin males (average 3.68 kg) are substantially larger than females (average 2.54 kg) [41], if strength is an important factor affecting variance in bite rates, adult males should have significantly higher bite rates than adults females. For this comparison, significantly higher return rates by males would suggest that either, a) strength has a large effect on foraging efficiency, or b) males are able to monopolize access to better (relatively easier-to-acquire) food items within each food species. Similar return rates for males and females could be the result of at least three different scenarios: 1) strength does not have a large effect on foraging efficiency, 2) strength does have a large effect on foraging efficiency but only up until a certain strength threshold which adult females have already attained, or 3) females and males achieve similar return rates through different methods with males taking advantage of strength and females taking advantage of skills. A second test was run to discern which of these scenarios is most likely.

In this second test I compared the return rates of adult and subadult females for difficult-to-acquire fruits. Subadult females (ages 5–7 years) have completed most of their growth [42] and are therefore likely to be nearly as strong as adult females, but do not begin to reproduce until age 7 on average [34]. Male capuchins, on the other hand, do not attain full body size until well after the subadult period and thus are less useful for trying to disentangle the roles of skill versus strength in determining return rates. If adult females have higher return rates for difficult-to-acquire fruits than do subadult females, it is likely that these differences stem largely from differences in skill rather than from differences in strength.

A third potential test, which would include both skill and strength as predictors of return rates, was not informative because the correlation between strength and skill levels was very high.

This project was approved by the University of New Mexico Office of Animal Care and Compliance (Protocol 07UNM068, Animal Welfare Assurance # A4023-01, USDA Registration # 85-R-0002).

## Results

A total of 608 fruit foraging focal follows across all age classes and food difficulty levels were included in the analysis to examine the effect of age and difficulty level on foraging return rates (Table 1). The average duration for focal follows was 4.7 minutes. 39 focal follows were eliminated from the analysis because either aggression or a displacement involving the focal individual occurred. 3705 behaviors were recorded during scan samples and time allocation budgets were calculated from these data. The percentage of time that each age class spent foraging for fruits ranged from 26–30% (Table 2). Within the fruit foraging activity budget, adults spent the most time foraging for difficult-to-acquire fruits while subadults spent the least amount of time on these fruits. Younger juveniles spent the most time foraging for easy to acquire fruits while adults spent the least amount of time (Table 2). Of the total 3705 scan behaviors observed, 459 involved some form of social encounter. Out of these 459 social behaviors, 17 involved either an aggression or a displacement.

**Table 1:** Number of Fruit Forage Focal Follows by Age Categories and Acquisition Difficulty Level.

Age Class	Easy	Medium	Difficult	Total
Adults	112	83	128	323
Subadults	46	33	20	99
Older Juveniles	33	33	27	93
Younger Juveniles	44	23	26	93
Totals	235	172	201	608

**Table 2:** Fruit Foraging Activity Budget.

	Proportion of activity budget spent:	Percent of fruit foraging time spent foraging for:		
Age Class	Foraging for Fruit	Difficult Fruits	Medium Fruits	Easy Fruits
Adults	27%	54	18	27
Subadults	27%	38	27	36
Older juveniles	30%	48	23	29
Younger juveniles	26%	41	27	33

Table 2 legend: vales in this table were calculated from scan sample data.

Return rates for difficult-, medium-, and easy-to-acquire fruits: For difficult-to-acquire, medium-to-acquire, and easy-to-acquire fruits, sex was not a significant factor in predicting return rates (difficult: χ^2^ = 2.966; p = 0.564, medium: χ^2^ = 0.728; p = 0.948, easy: χ^2^ = 6.715; p = 0.152) and therefore, for these analyses, sexes were combined. There were significant differences in return rates for difficult-to-acquire fruits between *adults* and all other age classes, with *adults* achieving significantly higher return rates (-2.41≥Z≥-2.67, p: 0.008–0.016, Table 3). For medium-to-acquire fruits there were no significant differences between adults and any other age class (-0.34≥Z≥0.98, p: 0.328–0.737, Table 3). For easy-to-acquire fruits, there were also no significant differences between adults and any other age class (-0.13≥Z≥1.72, p: 0.086–0.896, Table 3).

**Table 3:** Fruit Return Rates for Juvenile Age Classes Compared with Adults.

Age Class	Bites/Sec	Estimate	Std. Error	z value	Pr(>|z|)
	Difficulty Level = Difficult				
Adults	0.067	-2.71	0.29	N/A	N/A
Subadults	0.020	-1.20	0.45	-2.67	0.008
Older Juveniles	0.027	-0.90	0.37	-2.41	0.02
Younger Juveniles	0.026	-0.95	0.38	-2.52	0.01
	Difficulty Level = Medium				
Adults	0.19	-1.69	0.16	N/A	N/A
Subadults	0.23	0.24	0.24	0.98	0.33
Older Juveniles	0.17	-0.09	0.24	-0.38	0.71
Younger Juveniles	0.17	-0.09	0.28	-0.34	0.74
	Difficulty Level = Easy				
Adults	0.15	-1.90	0.22	N/A	N/A
Subadults	0.21	0.35	0.20	1.72	0.09
Older Juveniles	0.15	-0.03	0.24	-0.13	0.90
Younger Juveniles	0.18	0.20	0.21	0.97	0.33

For each model adults were the reference group. Data were split into food difficulty level subsets: difficult, medium, easy (top to bottom). Model = lmer(total number of bites ~ age class * sex + (1|focal subject ID) + (1|food item ID) + (1|obesrvation number) + offset(log(duration of observation)), family = Poisson). Bite rates are calculated as number of bites ingested divided by the total time it took an individual to forage for a food item.

Skill vs. strength—Adult males, adult females, and subadult females: Adult males did not have significantly different return rates from adult females for difficult-to-acquire fruits, rather adult male and adult females had nearly identical return rates for these fruits (Z = 0.03, p = 0.978, Table 4). Subadult females had substantially lower, however not quite significantly lower, return rates for difficult-to-acquire fruits than adults females (Z = -1.94, p = 0.052, Table 5).

**Table 4:** Difficult Fruit Return Rates for Adult Males Compared with the Adult Females.

	Difficulty Level = Difficult, Age Class = Adults				
Age Class	Bites/Sec	Estimate	Std. Error	z value	Pr(>|z|)
Adult Females	0.079	-2.54	0.31	N/A	N/A
Adult Males	0.079	0.01	0.35	0.03	0.978

For this model adult females were the reference group. Only difficult to acquire foods were included in this analysis. Model = lmer(total number of bites ~ age class * sex + (1|focal subject ID) + (1|food item ID) + (1|obesrvation number) + offset(log(duration of observation)), family = Poisson). Bite rates are calculated as number of bites ingested divided by the total time it took an individual to forage for a food item.

**Table 5:** Difficult Fruit Return Rates for Subadult Females Compared with the Adult Females.

	Difficulty Level = Difficult, Sex = Female				
Age Class	Bites/Sec	Estimate	Std. Error	z value	Pr(>|z|)
Adult Females	0.085	-2.47	0.27	N/A	N/A
Subadult Females	0.028	-1.09	0.56	-1.94	0.052

For this model adult females were the reference group. Only difficult to acquire foods were included in this analysis. Model = lmer(total number of bites ~ age class * sex + (1|focal subject ID) + (1|food item ID) + (1|obesrvation number) + offset(log(duration of observation)), family = Poisson). Bite rates are calculated as number of bites ingested divided by the total time it took an individual to forage for a food item.

## Discussion

In support of the first prediction for the difficult diet hypothesis, adults had significantly higher return rates than any of the younger age classes, including subadults, for fruits that were ranked as difficult-to-acquire. This indicates that either skill and/or strength is likely to affect foraging return rates for difficult-to-acquire fruits.

In support of the second prediction of the difficult diet hypothesis, three lines of evidence imply that variation in skill explains at least some of the observed variance in return rates for difficult-to-acquire fruits. First, no significant differences between age classes for easy-to-acquire fruits implies that when little strength and skill are required to obtain foods, individuals in different age classes are capable of achieving similar return rates.

Second, very similar return rates for adult males and adult females for difficult-to-acquire fruits, implies that strength does not explain a significant amount of the observed variation in return rates for these fruits, at least for individuals who have achieved the strength level of an adult female. It is not unlikely that males and females achieve these similar return rates through different means however, where, males may take advantage of their strength while females may rely more on skills. If this were the case, it would not change the general conclusion that skill level is an important factor influencing variation in return rates, however, it could mean that the acquisition of skill is less important for males.

Third, subadult females, who are nearly equal in size to adult females, but younger and therefore less experienced, had substantially lower return rates for difficult-to-acquire fruits compared with adults. Female subadults are likely to have similar strength to adult females, thus, this difference in return rates is likely to be, at least partially, the result of differences in skill.

Foraging ontogeny in capuchins has been studied by a number of different investigators and has resulted in somewhat different conclusions about the age at which these monkeys achieve foraging competency [11, 33, 43–45]. These findings could lead to different interpretations as to whether the prolonged juvenile period in capuchins may ultimately be the result of needing to learn foraging behaviors, the result of avoiding starvation in the face of competition [11], or the result of some other driving factor or factors. The results from this study help to clarify that the age at optimal foraging efficiency is expected to take a long time for foods that require the greatest degree of skill and/or knowledge to obtain and process, but only a short time for foods that require little skill and/or knowledge to obtain and process.

Support for the difficult diet version of the needing to learn hypothesis implies that capuchins are benefiting from investments into embodied capital in the form of fruit foraging skills. According to life history theory, energy that is used for one function is then unavailable for other functions [46], and thus these energy and time investments into learning foraging skills may divert energy away from physical growth, thereby lengthening the juvenile period.

There are several patterns in fruit return rates that deserve further discussion because they do not seem to fit well with the difficult diet hypothesis for this population of capuchins. The first is the pattern whereby return rates for difficult-to-acquire fruits do not appear to improve consistently within the juvenile period (they are not higher in subadults than in older juveniles). One explanation for this trend could be that difficult-to-acquire fruits require the entire juvenile period before detectable improvements in return rates are achieved. In other words, there may be a threshold of skill necessary to obtain efficient return rates for difficult-to-acquire foods. Another explanation could be that, pre-reproductive individuals that are younger, due to their low return rates, only forage for difficult-to-acquire fruit items in more favorable conditions. On the other hand, pre-reproductive individuals who are older, who should be more skilled at foraging, might take on difficult-to-acquire fruits in less favorable conditions (such as when they are rarer or fewer are ripe). This would result in lower average return rates for juveniles who are older, despite greater skill and strength. Future studies will be necessary to test these hypotheses.

The results from this study do not negate the possibility that additional factors proposed in other models may also account for some of the variation in age at first reproduction. For example, factors other than foraging complexity may select for larger brains, and brain growth may constrain the length of the juvenile period [5]. Another possibility is that factors including, but not limited to, foraging complexity may select for slow growth rates to decrease a juvenile’s risk of starvation, and thereby result in longer juvenile periods (ecological risk avoidance hypothesis) [11]. A major difference in the ecological risk avoidance hypothesis and the difficult diet hypothesis is that in the latter, foraging return rates are expected to correlate with age at first reproduction.

The methods outlined in this study for assigning difficulty levels to food items based on skill and strength requirements were convenient and likely informative. In the future, the addition of parametric variables, such as the average force required to break or puncture each food item, would add a degree of objectivity to these measurements. These types of measurements have been developed and used by several researchers (e.g., [47, 48]) to assess preferred versus fallback foods, and to help understand morphological variation in craniofacial morphology and food preferences. The methods developed in these papers could be used to assign quantitative values for strength requirements for various food processing steps.

Perhaps the biggest limitation to this study is lack of control for dominance status. Too few interactions involving aggression and/or displacements occurred over the study period to enable an accurate determination of ranks. In a study of white-faced capuchin monkeys in a different part of Costa Rica, Vogel [49] found that higher-ranked individuals have higher energy intake rates, whether or not aggression is actually observed in a particular feeding tree, for 78% of the tree species included in her study. At the same study site, Perry [50] found that males are almost always dominant in dyadic interactions between adult males and adult females. If both of these patterns are true for the capuchins in this study, it seems unlikely that dominance would explain all of the variation in fruit foraging return rates because if this were true, we would expect females to have lower return rates than males. Further studies are necessary to test whether age differences in foraging for difficult-to-acquire fruits would remain significant if rank was added to the models.

## Conclusions

The results from this study imply that skill and experience account for some of the observed variation in return rates for difficult fruits, that acquisition of fruit foraging skills entails a long learning phase in capuchins, that investments into embodied capital in the form of fruit foraging skills provide fitness benefits to capuchins, and that the needing-to-learn hypothesis should not be ruled out as a factor to help explain the adaptation of long juvenile periods in these genera (*Cebus and Sapujus*). These traits are likely to apply to other species that inhabit particularly complex foraging niches and also have long juvenile periods.

## Supporting Information

S1 File Fruit Focal Follow Data (Table A) and Scan Sample Data Used for This Study (Table B). (XLSX)

S1 Table Difficulty Level Assignments for the Top Ten Most Commonly Eaten Foods. S1 Table legend: Difficulty levels 1–3 followed by the description of the behaviors required for each foraging activity. Food items that are foraged from more than one location (i.e. ground or tree) are included multiple times to reflect difficulty levels for foraging from their respective locations.
(DOCX)

S2 Table Food Difficulty Levels for All Study Foods. S2 Table legend: food difficulty levels assigned as described in methods section of text.
(DOCX)
