# Supplementary material for: Ontogeny of Foraging Competence in Capuchin Monkeys (Cebus capucinus) for Easy versus Difficult to Acquire Fruits: A Test of the Needing to Learn Hypothesis
Source: PLoS One. 2015 Sep 15;10(9):e0138001. doi: 10.1371/journal.pone.0138001 (PMC4570712; doi:10.1371/journal.pone.0138001)
Supplement: S1 Table — legend: Difficulty levels 1–3 followed by the description of the behaviors required for each foraging activity. Food items that are foraged from more than one location (i.e. ground or tree) are included multiple times to reflect difficulty levels for foraging from their respective locations. (DOCX) [file pone.0138001.s002.docx]

Supplementary Table 1 for:

**Ontogeny of foraging competence in capuchin monkeys (Cebus capucinus) for easy versus difficult to acquire fruits: A test of the needing to learn hypothesis**

**S1 Table:** **Difficulty Level Assignments for the Top Ten Most Commonly Eaten Foods**

| **Strength or Skill** | **Food Item** | **Search Rank and Description** | | | **Harvest Rank and Description** | | **Process Rank and Description** | | |
| --- | --- | --- | --- | --- | --- | --- | --- | --- | --- |
| Strength | *Hampea appendiculata* | 1-unconcealed ripe fruits in tree canopy | | | 1-pluck from thin stem | | 1.5-pulp covered seeds from dehiscing fruits directly ingested, seeds spit out. Indehiscent fruits must be opened along seams of thick fleshy rind. | | |
| Skill |  | 1.5-Search abundant canopy fruits. **Smell** indehiscent fruits to determine ripeness. Dehiscent fruits are ripe | | | 1-unspecific tug | | 2-remove pulp-covered seeds from casing, closed fruits must be opened along seems, seeds are spit out after pulp is removed | | |
| Strength | *Genipa americana* (from ground) | 1-unconcealed fruits on ground under tree | | | 2-harvest heavy (avg. 250 g) fallen fruits from ground | | 2-remove thick, fleshy rind | | |
| Skill |  | 2-locate a ripe-rotten fruit by **touching** and/or **smelling** | | | 1-harvest fallen fruit from ground | | 2-remove thick, fleshy peel, extract pulp and spit out seeds. | | |
| Strength | *Tabemaemontana alba* | 1-unconcealed ripe fruits in tree canopy | | | 1-pluck from thin stem | | 2-remove thick, fleshy peel | | |
| Skill |  | 2-locate **rare** ripe (dehiscing) fruits in canopy | | | 1-unspecific tug | | 1-open fruits ready to ingest, spit out seeds. | | |
| Strength | *Eugenia sp. or Pimenta sp.* | 1-unconcealed ripe fruits in tree canopy | | | 1-pluck from thin stem | | 1-ready to ingest | | |
| Skill |  | 1-locate ripe fruit (dark purple and odoriferous) in canopy | | | 1-unspecific tug | | 1-ready to ingest, spit out seeds | | |
| Strength | *Morinda citrofolia* | 1-unconcealed ripe fruits in tree canopy | | | 1-pluck from thin stem | | 1-ready to ingest | | |
| Skill |  | 2-search canopy for **rare** ripe fruits (strongly odoriferous, soft) among many upripe fruits | | | 1-unspecific tug | | 1-ready to ingest, spit out seeds | | |
| Strength | *Elaeis oleifera* (from tree) | 1-unconcealed ripe fruits in infructescence | | | 3-forcefully pry from tightly packed infructescence | | 1-mostly ready to ingest, remove or spit out fibers while eating soft, oily pulp | | |
| Skill |  | 2-search large infructescence for the **rare** fruit that is loose enough to pry from the cluster | | | 3-position body and hands to get maximum force and leverage while navigating spiky infructescence | | 2-removal of thick fibers from soft pulp | | |
| Strength | *Carludovica rotundifolia* | 1-unconcealed fruits on stalks | | | 1-pluck from tiny thin stem | | 1-ready to ingest | | |
| Skill |  | 1-search stalks for ripe fruits (bright orange) | | | 1-unspecific tug | | 1-ready to ingest | | |
| Strength | *Terminalia catappa* | 1-unconcealed ripe fruits in tree canopy | | | 1-pluck from thin stem | | 1.5-mostly ready to ingest, occassionaly pound on hard surface to soften pulp | | |
| Skill |  | 2-locate **rare** ripe (yellow or pink, odoriferous, soft) fruits among many unripe fruits | | | 1-unspecific tug | | 1.5-ready to ingest, occasionally pound process to soften pulp. | | |
| Strength | *Genipa americana* (unopen in tree) | 1-unconcealed ripe fruits in tree canopy | | | 2-detach from thick stem | | 2-remove thick, fleshy peel | | |
| Skill |  | 2-Locate a **rare** ripe fruit (softer, odoriferous) by **smelling** and **squeezing**. Many fruits are in the canopy at one time with the vast majority unripe. | | | 2-well-directed tug, may require twisting to weaken thick stem | | 2-Remove a portion of the thick, fleshy rind | | |
| Strength | *Genipa americana* (open in tree) | 1-unconcealed ripe fruits in tree canopy | | | 1-harvest pulp directly from open ripe fruit | | 1-ready to ingest | | |
| Skill |  | 1.5-locate a **rare** open fruit (likely to be ripe if already partially eaten). | | | 1-harvest pulp directly from fruit | | 1-ready to ingest | | |
| Strength | *Elaeis oleifera* (from the ground) | 1-unconcealed ripe fruits in infructescence | | | 1-harvest fallen fruits from ground | | 1-mostly ready to ingest, can remove thin, papery peel, remove or spit out fibers imbedded in soft, oily pulp. | | |
| Skill |  | 1-search ground under tree for partially eaten fruits | | | 1-harvest fallen fruit from ground | | 1-Ready to ingest, fibers removed during ingestion. | | |
| **Food Item** | | | **Total Strength** | **Total Skill** | | **Total Difficulty** | | **Difficulty Level** |  |
| *Hampea appendiculata* | | | 3.5 | 4.5 | | 8 | | Medium |  |
| *Genipa Americana* (from ground) | | | 5 | 5 | | 10 | | Difficult |  |
| *Tabemaemontana alba* | | | 4 | 4 | | 8 | | Medium |  |
| *Eugenia sp. or Pimenta sp.* | | | 3 | 3 | | 6 | | Easy |  |
| *Morinda citrofolia* | | | 3 | 4 | | 7 | | Easy |  |
| *Elaeis oleifera* (from tree) | | | 5 | 7 | | 12 | | Difficult |  |
| *Carludovica rotundifolia* | | | 3 | 3 | | 6 | | Easy |  |
| *Terminalia catappa* | | | 3.5 | 4.5 | | 8 | | Medium |  |
| *Genipa Americana* (unopen on tree) | | | 5 | 6 | | 11 | | Difficult |  |
| *Genipa Americana* (open on tree) | | | 3 | 3.5 | | 6.5 | | Easy |  |
| *Elaeis oleifera* (from ground) | | | 3 | 3 | | 6 | | Easy |  |

S1 Table legend: Difficulty levels 1-3 followed by the description of the behaviors required for each foraging activity. Food items that are foraged from more than one location (i.e. ground or tree) are included multiple times to reflect difficulty levels for foraging from their respective locations.
