# Supplementary material for: Ontogeny of Foraging Competence in Capuchin Monkeys (Cebus capucinus) for Easy versus Difficult to Acquire Fruits: A Test of the Needing to Learn Hypothesis
Source: PLoS One. 2015 Sep 15;10(9):e0138001. doi: 10.1371/journal.pone.0138001 (PMC4570712; doi:10.1371/journal.pone.0138001)
Supplement: S2 Table — legend: food difficulty levels assigned as described in methods section of text. (DOCX) [file pone.0138001.s003.docx]

Supplementary Table 2 for:

**Ontogeny of foraging competence in capuchin monkeys (Cebus capucinus) for easy versus difficult to acquire fruits: A test of the needing to learn hypothesis**

**S2 Table:** **Food Difficulty Levels for All Study Foods**

|  | **Strength** | | | | **Skill** | | | |  |  |
| --- | --- | --- | --- | --- | --- | --- | --- | --- | --- | --- |
| **Food item** | **Search** | **Harvest** | **Process** | **Total** | **Search** | **Harvest** | **Process** | **Total** | **Total Difficulty** | **Difficulty Level** |
| *Hampea appendiculata* | 1 | 1 | 1.5 | 3.5 | 1.5 | 1 | 2 | 4.5 | 8 | Medium |
| *Herrania purpurea* | 1 | 2 | 3 | 6 | 3 | 2 | 2 | 7 | 13 | Difficult |
| *Chrysophyllum cainito* | 1 | 1 | 2 | 4 | 1 | 1 | 2 | 4 | 8 | Medium |
| *Prioria copaifera* | 1 | 1 | 3 | 5 | 3 | 1 | 3 | 7 | 12 | Difficult |
| *Ficus insipida* | 1 | 1 | 1 | 3 | 1.5 | 1 | 1 | 3.5 | 6.5 | Easy |
| *Inga vera subsp. vera* | 1 | 1 | 2 | 4 | 2 | 1 | 2 | 5 | 9 | Medium |
| *Genipa Americana* (from ground) | 1 | 2 | 2 | 5 | 2 | 1 | 2 | 5 | 10 | Difficult |
| *Psidium guajava* | 1 | 1 | 1 | 3 | 1 | 1 | 1 | 3 | 6 | Easy |
| *Posoqueria latifolia* | 1 | 1 | 2 | 4 | 2 | 1 | 2.5 | 5.5 | 9.5 | Medium |
| *Siparuna sp.* | 1 | 1 | 1.5 | 3.5 | 1 | 1 | 2 | 4 | 7.5 | Medium |
| *Tabemaemontana alba* | 1 | 1 | 2 | 4 | 2 | 1 | 1 | 4 | 8 | Medium |
| *Amphitecna latifolia* | 1 | 1 | 3 | 5 | 3 | 1 | 3 | 7 | 12 | Difficult |
| *Spondias mombin* | 1 | 1 | 1 | 3 | 2 | 1 | 1 | 4 | 7 | Easy |
| Lemon gatorade berries (unknown species) | 1 | 1 | 1 | 3 | 1 | 1 | 1 | 3 | 6 | Easy |
| *Neea sp.* | 1 | 1 | 1 | 3 | 1 | 1 | 1 | 3 | 6 | Easy |
| *Morinda citrofolia* | 1 | 1 | 1 | 3 | 2 | 1 | 1 | 4 | 7 | Easy |
| *Elaeis oleifera* (from tree) | 1 | 3 | 1 | 5 | 2 | 3 | 2 | 7 | 12 | Difficult |
| *Vitis tiliifolia* | 1 | 1 | 1 | 3 | 1 | 1 | 1 | 3 | 6 | Easy |
| Palm heart (*Aracaceae* family) | 1 | 3 | 1 | 5 | 2 | 3 | 1 | 6 | 11 | Difficult |
| *Attalea rostrata* | 1 | 2 | 3 | 6 | 2 | 2 | 2.5 | 6.5 | 12.5 | Difficult |
| *Carludovica rotundifolia* | 1 | 1 | 1 | 3 | 1 | 1 | 1 | 3 | 6 | Easy |
| *Bactris gasipeas* | 1 | 2 | 1 | 4 | 2 | 1 | 1 | 4 | 8 | Medium |
| *Piper sp.* | 1 | 1 | 1 | 3 | 2 | 1 | 1 | 4 | 7 | Easy |
| *Psychotria marginata* | 1 | 1 | 1 | 3 | 1 | 1 | 1 | 3 | 6 | Easy |
| *Terminalia catappa* | 1 | 1 | 1.5 | 3.5 | 2 | 1 | 1.5 | 4.5 | 8 | Medium |
| *Raphia taedigera* | 1 | 1 | 3 | 5 | 2 | 2 | 3 | 7 | 12 | Difficult |
| *Genipa Americana* (from tree) | 1 | 2 | 2 | 5 | 2 | 2 | 2 | 6 | 11 | Difficult |
| *Elaeis oleifera* (from ground) | 1 | 1 | 1 | 3 | 1 | 1 | 1 | 3 | 6 | Easy |
| surface insect | 1 | 1 | 1 | 3 | 1 | 2 | 1 | 4 | 7 | Easy |
| embedded insect | 3 | 3 | 1 | 7 | 3 | 2 | 1 | 6 | 13 | Difficult |
| *Conostegia sp.* | 1 | 1 | 1 | 3 | 1 | 1 | 1 | 3 | 6 | Easy |
| *Cocos nucifera* | 1 | 2 | 3 | 6 | 2 | 2 | 3 | 7 | 13 | Difficult |
| *Musa acuminata* | 1 | 2 | 2 | 5 | 2 | 1 | 2 | 5 | 10 | Difficult |
| Bejuco de agua. (unknown species) | 1 | 1 | 1 | 3 | 1 | 1 | 1.5 | 3.5 | 6.5 | Easy |
| Berries (unknown species) | 1 | 1 | 1 | 3 | 1 | 1 | 1 | 3 | 6 | Easy |
| *Monostera Deliciosa* | 1 | 2 | 1 | 4 | 2 | 2 | 1 | 5 | 9 | Medium |
| *Musa acuminata* | 1 | 3 | 1 | 5 | 1 | 2 | 1 | 4 | 9 | Medium |
| *Pentagonia monocaulis* | 1 | 1 | 1 | 3 | 1 | 1 | 1 | 3 | 6 | Easy |
| *Snails* (unknown species) | 1 | 1 | 3 | 5 | 2 | 1 | 3 | 6 | 11 | Difficult |
| *Dialium sp.* | 1 | 1 | 1 | 3 | 1 | 1 | 2 | 4 | 7 | Easy |
| *Alibertia edulis* | 1 | 1 | 1 | 3 | 1 | 2 | 2 | 5 | 8 | Medium |
| *Cocos nucifera* | 1 | 2 | 2 | 5 | 1 | 2 | 2 | 5 | 10 | Difficult |

S2 Table legend: food difficulty levels assigned as described in methods section of text.
